# P-1259. Transferring knowledge from gentamicin pharmacokinetics in neonates to other aminoglycosides

**DOI:** 10.1093/ofid/ofae631.1441

**Published:** 2025-01-29

**Authors:** Tiffany Lee, Jasmine Hughes, Nicole M Hall, Matthew L Brown, William S Edwards, Jon Faldasz

**Affiliations:** InsightRX, Philadelphia, Pennsylvania; InsightRX, Philadelphia, Pennsylvania; UAB Hospital, Birmingham, Alabama; University of Alabama at Birmingham, Birmingham, Alabama; University of Alabama at Birmingham, Birmingham, Alabama; InsightRX, Philadelphia, Pennsylvania

## Abstract

**Background:**

Gentamicin (GM) is frequently prescribed in the neonatal population, and its pharmacokinetics (PK) in this population are therefore well-studied. However, recent findings of diminished GM efficacy against *Pseudomonas* spp. have prompted increased use of other aminoglycosides with less well-defined PK, which poses a challenge for model-informed precision dosing (MIPD). Although ultimately the goal for dose individualization will be drug-specific models for neonatal patients, as an interim measure, this study investigates the performance of GM models in neonates receiving amikacin (AK) and tobramycin (TO).

**Figure:**
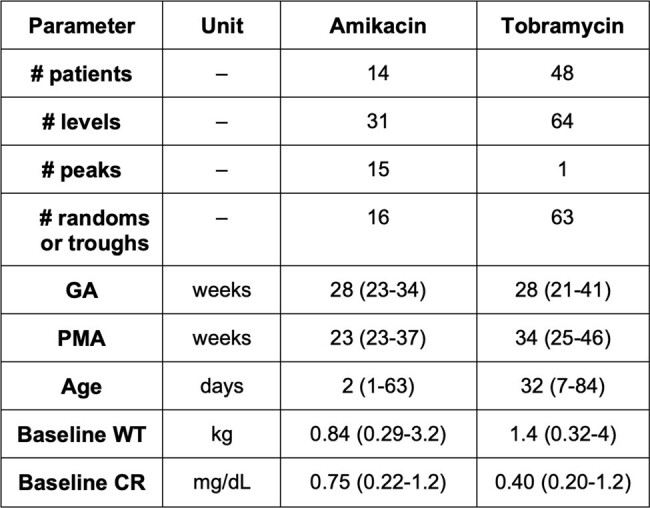

Patient Characteristics. Numbers are represented as counts or median (range) as appropriate. GA: gestational age; PMA: post-menstrual age; WT: weight; CR: creatinine.

**Methods:**

Neonatal patient data entered into the InsightRX Nova model-informed precision dosing (MIPD) platform during routine clinical care were de-identified and analyzed retrospectively. Patients were included if their postnatal age was < 90 days or their postmenstrual age was < 44 weeks, received at least one dose of AK or TO and provided at least one therapeutic drug monitoring sample (TDM) within 48 hours of an administered dose. For each patient and for each model, samples were grouped by dosing interval and iteratively used to predict future levels using Bayesian forecasting, mimicking clinical implementation of MIPD. Prediction error was assessed using root mean square error (RMSE), mean percent error (MPE), and Accuracy, defined as percent of samples within 20% of predicted values or correctly identifying a trough under 1 mg/L.

**Figure:**
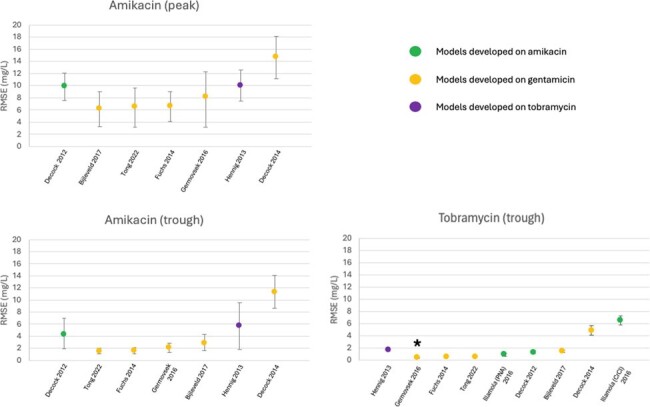

RMSE for aminoglycoside models developed on amikacin, gentamicin or tobramycin data sets and evaluated on clinical amikacin and tobramycin data sets. The best native performing model for each clinical data set serves as the reference model & is listed first, with subsequent models ordered by best to worst performance. Asterisks denotes statistical significance relative to the reference model.

**Results:**

Overall, 14 AK courses with 31 AK TDMs, and 48 TO courses with 63 TO TDMs were included in the analysis (Table 1). RMSE, MPE, and Accuracy are displayed in Figures 1-3. Data on TO peaks were excluded due to low sample size. Of the AK courses, most GM models outperformed available AK models for both peak and trough levels. Of the TO courses, GM models exhibited significantly improved RMSE and MPE values, as well as significantly higher rates of accuracy when predicting trough levels compared to those of the available TO model.

**Figure:**
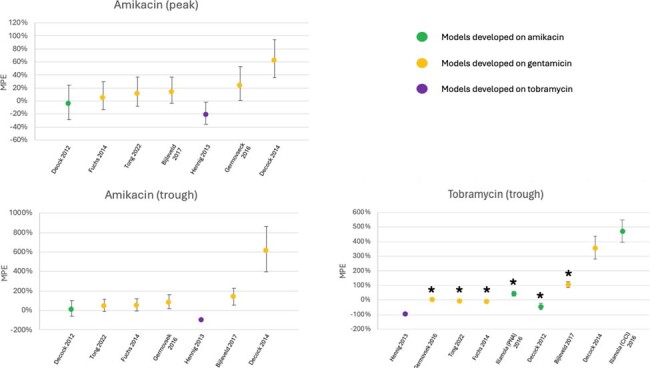

Mean percent error for aminoglycoside models developed on amikacin, gentamicin or tobramycin data sets and evaluated on clinical amikacin and tobramycin data sets. The best native performing model for each clinical data set serves as the reference model & is listed first, with subsequent models ordered by best to worst performance. Asterisks denotes statistical significance relative to the reference model.

**Conclusion:**

PK models developed in neonates receiving GM match or outperform drug-specific models in predicting AK and TO exposures in neonates. Further research is needed to better understand model performance for TO peak levels in neonates and clinical implications of cross-module model use.

**Figure:**
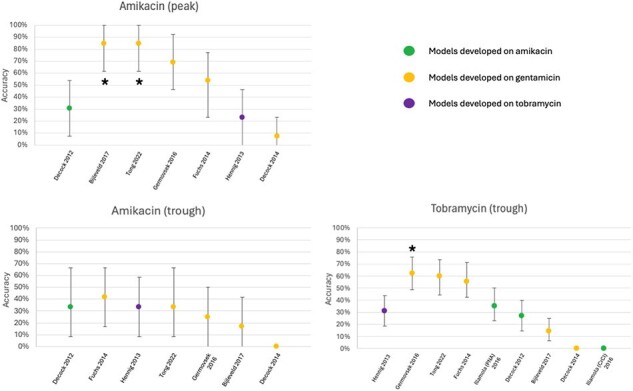

Accuracy for aminoglycoside models developed on amikacin, gentamicin or tobramycin data sets and evaluated on clinical amikacin and tobramycin data sets. The best native performing model for each clinical data set serves as the reference model & is listed first, with subsequent models ordered by best to worst performance. Asterisks denotes statistical significance relative to the reference model.

**Disclosures:**

**Tiffany Lee, PharmD**, InsightRX: Employee|InsightRX: Stocks/Bonds (Private Company) **Jasmine Hughes, PhD**, InsightRX: Employee|InsightRX: Stocks/Bonds (Private Company) **Jon Faldasz, PharmD, BCPS**, InsightRX: Employee|InsightRX: Stocks/Bonds (Private Company)

